# Competency-based learning in an ambulatory care setting: Implementation of simulation training in the Ambulatory Care Rotation during the final year of the MaReCuM model curriculum

**DOI:** 10.3205/zma001153

**Published:** 2018-02-15

**Authors:** Martin Dusch, Elisabeth Narciß, Renate Strohmer, Katrin Schüttpelz-Brauns

**Affiliations:** 1 Hannover Medical School, Department of Anaesthesia and Critical Care Medicine, Hannover, Germany; 2 Medical Faculty Mannheim Heidelberg University, University Medicine Mannheim (UMM), Mannheim, Germany

**Keywords:** ambulatory care, competency-based education, medical education, outpatient care, patient simulation

## Abstract

**Aim: **As part of the MaReCuM model curriculum at Medical Faculty Mannheim Heidelberg University, a final year rotation in ambulatory care was implemented and augmented to include ambulatory care simulation. In this paper we describe this ambulatory care simulation, the designated competency-based learning objectives, and evaluate the educational effect of the ambulatory care simulation training.

**Method: **Seventy-five final year medical students participated in the survey (response rate: 83%). The control group completed the ambulatory rotation prior to the implementation of the ambulatory care simulation. The experimental group was required to participate in the simulation at the beginning of the final year rotation in ambulatory care. A survey of both groups was conducted at the beginning and at the end of the rotation. The learning objectives were taken from the National Competency-based Catalogue of Learning Objectives for Undergraduate Medical Education (NKLM).

**Results: **The ambulatory care simulation had no measurable influence on students’ subjectively perceived learning progress, the evaluation of the ambulatory care rotation, or working in an ambulatory care setting. At the end of the rotation participants in both groups reported having gained better insight into treating outpatients. At the beginning of the rotation members of both groups assessed their competencies to be at the same level. The simulated ambulatory scenarios were evaluated by the participating students as being well structured and easy to understand. The scenarios successfully created a sense of time pressure for those confronted with them. The ability to correctly fill out a narcotic prescription form as required was rated significantly higher by those who participated in the simulation. Participation in the ambulatory care simulation had no effect on the other competencies covered by the survey.

**Discussion:** The effect of the four instructional units comprising the ambulatory care simulation was not measurable due to the current form or the measurement point at the end of the 12-week rotation. The reasons for this could be the many and statistically elusive factors relevant to the individual and the wide variety among final year rotation placements, the late point in time of the final survey, and the selection of simulated scenarios. The course is slated to undergo specific further development and should be supplemented with additional learning opportunities to ensure that the main learning objectives are covered. The description of the teaching format is meant to contribute to the ongoing development of medical education with an emphasis on competency in the areas of ambulatory care, communication, prevention and health promotion.

## Zusammenfassung

**Zielsetzung:** Im Rahmen des Modellstudiengangs MaReCuM der Medizinischen Fakultät Mannheim der Universität Heidelberg wurde das PJ-Quartal Ambulante Medizin implementiert und um die Lehrveranstaltung „Ambulanzsimulation“ erweitert. Die Ziele der Arbeit umfassen die Beschreibung der Lehrveranstaltung und der formulierten kompetenzbasierten Lernziele sowie die Untersuchung des Lerneffekts der Lehrveranstaltung.

**Methodik: **75 PJ-Studierende haben sich an der Fragebogenerhebung beteiligt (Rücklauf: 83%). Die Kontrollgruppe absolvierte das Quartal Ambulante Medizin vor Einführung der Lehrveranstaltung „Ambulanzsimulation“. Die Versuchsgruppe nahm verpflichtend an dieser Lehrveranstaltung zu Beginn des PJ-Quartals Ambulante Medizin teil. In beiden Gruppen erfolgte jeweils am ersten Tag des Quartals sowie 21 Tage vor Ende des Quartals eine Fragebogenerhebung. Die Lernziele wurden dem NKLM entnommen.

**Ergebnisse: **Die „Ambulanzsimulation“ hatte keinen messbaren Einfluss auf den subjektiv wahrgenommenen Lernerfolg, die Bewertung des Quartals Ambulante Medizin sowie das Arbeiten in einem ambulanten Setting. Die Teilnehmer beider Gruppen gaben am Ende des Quartals an, einen besseren Einblick in die Behandlung ambulanter Patienten gewonnen zu haben. Die Teilnehmer beider Gruppen schätzten die untersuchten Kompetenzen zu Beginn des Quartals gleich ein. Die simulierten Patientenfälle wurden durch die Teilnehmer als eher gut strukturiert und leicht verständlich bewertet. Die Szenarien waren geeignet, beim Bearbeiten Zeitdruck entstehen zu lassen. Die Kompetenz, ein BTM-Rezept den formalen Vorgaben entsprechend korrekt auszufüllen, schätzten die Teilnehmer der „Ambulanzsimulation“ signifikant besser ein. Auf die übrigen erhobenen Kompetenzen hatte die Teilnahme an der Ambulanzsimulation keinen Effekt.

**Diskussion: **Der Effekt der nur 4 Unterrichtseinheiten umfassenden Lehrveranstaltung „Ambulanzsimulation“ war in der untersuchten Form und am Ende des 12-wöchigen Quartals nicht messbar. Gründe dafür könnten die vielfältigen und statistisch nicht zu kontrollierenden Einflussfaktoren im Bereich der individuellen und sehr unterschiedlichen PJ-Rotationsplätze, der späte Zeitpunkt der Abschlusserhebung sowie die Auswahl der Simulationsszenarien sein. Die Lehrveranstaltung sollte spezifisch weiterentwickelt und möglicherweise um weitere Lerngelegenheiten ergänzt werden, um rotationsplatzübergreifend die Vermittlung der übergeordneten Lernziele sicher zu stellen. Die Beschreibung des Lehrformats versteht sich weiterhin als Diskussionsbeitrag im Rahmen der Weiterentwicklung des Medizinstudiums mit einer Stärkung der Kompetenzen im Bereich der ambulanten Patientenversorgung, der Kommunikation, der Prävention und Gesundheitsförderung.

## Introduction

In healthcare the majority of doctor-patient interactions now occur in an ambulatory setting [1]. This is not only desirable from a political perspective – outpatient before inpatient – but also possible due to progress in modern medicine [2]. Even at our own university hospital, many more patients receive care as outpatients than inpatients (219,834 [80%] outpatient versus 54,340 [20%] inpatient) [3]. Since medical education primarily deals with inpatients, outpatients who present common and important clinical problems or who display chronic disease progression are missing from the education of future physicians. For this reason, English-speaking countries recognized the need to focus on experience in primary care and teaching medicine in ambulatory settings [4]. In a 1995 review, David M. Irby elaborated the specifics of learning in an ambulatory setting [5]: The spectrum of patient cases is very diverse depending on the ambulatory care facility. Despite this, the experiences of the medical students in the ambulatory setting are still very comparable. This applies mainly to the following common characteristics and challenges of this particular area of medical practice: time pressure in providing patient care and a quick succession of patients [5]. In addition, we have identified further aspects in our own ambulatory care setting which are summarized in table 1 (Tab. 1).

Furthermore, the ambulatory setting provides the opportunity of long-term observation of the course of the disease over the timeframe of repeated visits. Thus it is possible to teach particularly sustainable healthcare strategies and preventive care as well as structured patient management during these visits. This also applies to dealing with social, financial and ethical aspects of disease [4], [6], [7], [8], [9], [10], [11], [12].

From the perspective of the teacher, time pressure and conflicting goals arise from the brief amounts of time allotted to ambulatory care appointments, especially since patients are usually still waiting for the actual consultation when preliminarily discussing their case with the medical student. Frequently there is neither direct observation of the interaction between medical student and outpatient, nor any probing for prior knowledge or providing with adequate feedback. Teaching in doctor’s offices usually allows a higher degree of exchange, feedback and supervision [5]. From the student’s point of view there are also deficits; Irby reports that final year medical students had difficulties recording the problems reported by the patient, that questions about adherence were not raised and important findings omitted during the presentation of the patient case [5].

How, despite the limitations of the learning environment, can ambulatory care medicine be made more accessible to medical students? The possibility of changing the system and allowing more time for student supervision and feedback in real-life situations is extremely limited.

By dividing the final year of medical undergraduate study (practical year) into quarters, a new rotation in ambulatory care was added to the MaReCuM model curriculum offered at the Medical Faculty Mannheim Heidelberg University. The aim was to address the limitations and conflicting learning objectives found in the ambulatory care setting. The ambulatory care simulation was introduced to provide a safe environment to better prepare the students for work in the ambulatory care setting. The learning objectives for the ambulatory care simulation focused on the nature of providing outpatient care and on the physician’s roles that are particularly important (communicator, health advocate, manager, professional). Including the ambulatory care setting in formal medical education has already been called for by other educators [13]. In this paper we describe and evaluate this new competency-based teaching format.

## Project description

### Learning objectives for the ambulatory care simulation

Four main learning objectives were identified. Students are able to:

1. recognize life-threatening, avoidable conditions and apply strategies to avert them;
2. integrate health promotion and prevention as basic elements of medical care;
3. apply the principles of medical documentation in a manner appropriate to the situation;
4. communicate appropriately with other health professions using medical terminology.

#### Simulation of patient interaction in the ambulatory care setting (ambulatory care simulation)

Based on the main learning objectives, a subset of more in-depth objectives was tailored to the content of the simulation scenarios. An example is presented in table 2 (Tab. 2).

The simulation of outpatient interaction took place at the skills lab (TheSiMa) of the Medical Faculty Mannheim. The rooms served as consultation rooms with a desk, telephone and documentation system. Each scenario shared these features of ambulatory care:

1. time constraint,
2. a complaint presented by a simulated patient (SP) which needed to be solved in real time,
3. a summary of the consultation by the doctor for the SP focusing on the next step or procedure.

**Figure 1:**
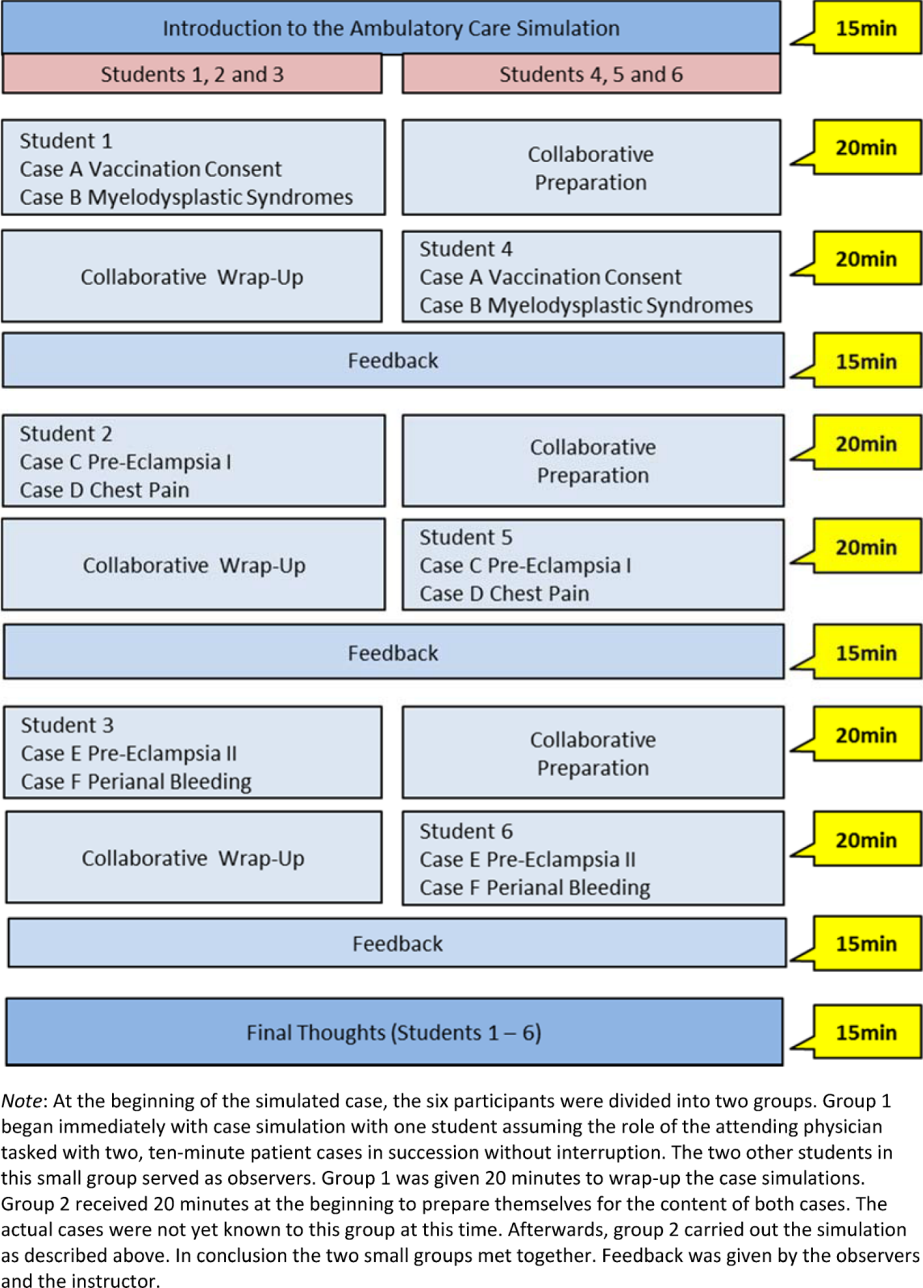
Schematic diagram of the ambulatory care simulation

Distractors were included into each scenario, for instance, one scenario included an accompanying family member. These ancillary details were meant to increase the level of stress and sense of time pressure. An example is presented in table 2 (Tab. 2). The scenarios were created in cooperation with colleagues from several medical disciplines. The ambulatory care simulation was conducted as small-group instruction with six students in each group. The simulated exercises encompassed four instructional units. At some point each participant assumed the role of the physician, while the others served as observers. Figure 1 (Fig. 1) provides an example of the procedure: two cases are attended to without any breaks or intervening discussion. Feedback was provided afterwards.

#### Training the simulated patients (SP)

Eighteen simulated patients were trained specifically for the cases selected for the ambulatory care simulation. The actors were recruited and trained using the preexisting SP program at the Medical Faculty Mannheim. Detailed scripts were written for the roles, including detailed descriptions of the symptoms, case histories and the patient (age, sex, behavior, questions and possible answers).

#### Research Questions

To evaluate this competency-based teaching format for effectiveness regarding the intended learning objectives, the following questions were examined:

- How do students evaluate this teaching format?
- Did participation in the ambulatory care simulation influence students’ subjectively perceived learning?
- Did participation in the ambulatory care simulation have an influence on the acquisition of competencies during the ambulatory care rotation?
- Did participation in the ambulatory care simulation have an influence on the evaluation of the ambulatory care rotation?
- Did participation in the ambulatory care simulation have an influence on working in the ambulatory care setting?

## Methods

### Sample

A total of 75 final year medical students participated in the survey (response rate: 83%). The control group (CG) encompassed students in the ambulatory care rotation prior to the introduction of the ambulatory care simulation; these students were recruited from the cohort for whom the final year started in August 2013. The experimental group (EG) consisted of students in the ambulatory care rotation after introduction of the mandatory ambulatory care simulation; this cohort began the final year in May 2014. In the CG there were 27 students in each of the two rotations prior to implementation of the ambulatory care simulation (14 women (54%) and 12 men (46%), 1 person without any indication of sex; average age 27.08 years (SD=3.16)). The EG was required to attend the ambulatory care simulation as part of final year courses (also two ambulatory care rotations in total) and had 48 participants (23 women (55%) and 19 men (45%), 6 without indication of sex; average age 27.36 years (SD=4.11)). No differences could be determined between CG and EG regarding distribution of sex (p=0.94 [likelihood ratio test]) or age (T(65)=-0.29; P=0.77).

#### Material

To evaluate the teaching format ambulatory care simulation, we created a questionnaire to serve as a pre-measure prior to the teaching intervention and a second questionnaire as a post-measure after the intervention. Both questionnaires contained multiple groups of questions with items to be rated on a six-point Likert scale (1=completely agree; 6=completely disagree). The organizational structure of both questionnaires, including all of the question groups, is shown in table 3 (Tab. 3).

In the group of questions *evaluating the simulated ambulatory care cases*, there were seven pre-formulated statements generally assessing the structure and difficulty of the simulated cases. One example item reads: “The cases are easy to understand.” (see table 4 (Tab. 4)).

In the group of questions *evaluating the ambulatory care simulation*, it was possible to generally assess the teaching intervention with 10 questions from the intervention-success inventory described by Kauffeld, Brennecke & Strack (2009) [14]. This questionnaire is based on the four levels of the Kirkpatrick pyramid. Two questions from the original questionnaire relating to the outcome level were not used. The word “training” was replaced with the term “ambulatory care simulation.” One example item reads: “I learned a lot of new information during the ambulatory care simulation.” (see table 5 (Tab. 5)).

In the group of questions on *subjectively perceived learning*, the learning success at the end of the ambulatory care rotation was covered by seven items for CG and nine for EG based on [15] (see table 6 (Tab. 6)).

In the group of questions addressing the *NKLM learning objectives*, the participants were asked to subjectively rate their own level of competence in 29 sub-competencies taken from the National Competency-based Catalogue of Learning Objectives for Undergraduate Medical Education (NKLM) relating to the learning objectives defined above for this project (see attachment 1 ).

In the group of questions *evaluating the ambulatory care rotation,* there were six pre-formulated statements generally assessing the rotation. One example item reads: “I learned a lot of new information during the ambulatory care rotation.” (see table 7 (Tab. 7)).

In the group of questions about *working in the ambulatory care setting*, the participants were able to rate their attitudes toward and expectations for working in the ambulatory care setting based on six pre-formulated statements. One example item reads: “I can well imagine working in an ambulatory care setting or in a doctor’s office as a professional goal.” (see table 8 (Tab. 8)).

#### Implementation

The undergraduate studies committee at the Medical Faculty Mannheim approved the implementation of the ambulatory care simulation for the final year rotation in ambulatory care. All participating students were informed of the study objectives and gave their written consent. The ambulatory care simulation was offered in small groups to all students within the first 14 days of the rotation.

The pre-measurement was taken at the introductory session on the ambulatory care rotation on the third day of the rotation. As part of the introductory session, the concept of the rotation was explained to the students, as was the ambulatory care simulation to the EG. Student questions were answered. The voluntary participants in both groups gave information such as age, sex, practical experience, and level of proficiency on the NKLM learning objectives.

The post-measurement took place during the final 21 days of the rotation. This was done so that even students who would be absent at the very end due to a rotation abroad or studying for exams would still have the opportunity to take the survey. All participants were asked about working in the ambulatory care setting, their evaluation of the rotation in ambulatory care, their subjectively perceived learning progress, and their subjective level of resulting proficiency regarding the NKLM learning objectives. The participants in the experimental group also responded to additional questions to evaluate the ambulatory care simulation. The evaluation procedure is shown in table 3 (Tab. 3).

#### Statistical analysis

Scores were calculated for the questionnaires with a reliability of α>0.70. Differences between the CG and EG before and after the rotation in ambulatory care were compared using two-way analysis of variance with repeated measures. The post-measurement differences between the CG and EG were analyzed with a one-way analysis of variance. In the results of the two-way analyses of variance with repeated measures each shows three values. The first, F_Time_, indicates if the values change over time. This shows the pre-post comparisons. The second value, F_T*Gr_, indicates if there is interaction between time and group. If this is the case, then it can be assumed that the intervention was effective. The third value, F_Group_, indicates if there is a difference between the groups, regardless of the time. In addition to test size and p value, η^2^ was also shown as effect size in the variance analyses. According to Cohen (1988), η^2^<0.0099 means there is no effect, η^2^≥0.0099 a small effect, η^2^≥0.0588 a moderate effect, and η^2^≥0.1379 a large effect [11], [16]. In addition to the scores, the individual items were also compared. To avoid the alpha error accumulation occurring with simultaneously applied significance tests, Bonferroni’s alpha adjustment was performed for the individual item analysis. The values are shown in the tables appearing in the results section (see table 6 (Tab. 6), table 7 (Tab. 7), table 8 (Tab. 8) and attachment 1 ).

## Results

### Evaluation of the teaching format

The simulated cases were not rated as being too easy. They were viewed as being mostly well structured, easy to understand and overall worked out (see table 4 (Tab. 4)). The reliability of the questions evaluating the cases in the sample is α=0.32.

The students are very likely to have positive memories of the ambulatory care simulation. Application in practice (transfer to routine work, increased satisfaction with work, and improvement of performance as a result of attending the ambulatory care simulation) was rated more negatively in comparison (see table 5 (Tab. 5)). The reliability of the questions asking for an evaluation of the ambulatory care simulation was α=0.90 for this sample.

#### Subjectively perceived learning

No difference in subjective perception of learning was seen between the EG (M_EG_=2.5; SD_EG_=0.7) and the CG (M_CG_=2.8; SD_CG_=0.7) with F(1)=2.38; p=0.13; η^2^=0.05. The results of the individual item analysis are presented in table 6 (Tab. 6). No differences could be detected among these either. The reliability of the questions on perceived learning is α=0.76.

#### NKLM learning objectives – Acquisition of competencies

The mean values for self-assessed competency revealed a wide dispersion in both groups between the highest assessment (1.5) and the lowest (4.4). Eight of the 29 competencies covered by the questions are in the upper tercile of the highest assessment (mean value 1.5 -2.5). These competencies were predominantly associated with the role of communicator. Seventeen of the 29 competencies are found in the middle tercile of the self-assessment (mean value 2.5 – 3.5). These competencies could not be assigned to a specific competency area. Four of the 29 competencies are seen in the lower tercile of the poorest assessments (mean value 3.5-4.5). These competencies were associated with resource allocation and quality management. Both groups measurably acquired more competencies at the end of the ambulatory care rotation (pre-measure: M_CG_=2.5, SD_CG_=0.7, M_EG_=3.0, SD_EG_=0.5; post-measure: M_CG_=2.3, SD_CG_=0.6, M_EG_=2.6, SD_EG_=0.5) with F_Time_(1)= 12.83; p<0.05; η^2^=0.24. Although both groups differed significantly: F_Group_(1)= 4.99; p=0.03; η^2^=0.11, this is not a result of attending the ambulatory care simulation: F_Time*Group_(1)= 0.42; p=0.52; η^2^=0.01. In terms of the individual items, the experimental group only showed a higher level of self-assessment connected with filling in narcotic prescription forms, a process that was explained during the ambulatory care simulation. Both groups rated their competencies in taking a patient history, prescribing medications, presenting cases, determining incapacity to work, and medical documentation as being higher at the end of the rotation than at the beginning. The results of the individual item analysis are included in the attachment 1 . The reliability of the questions about the NKLM learning objectives is α=0.93. 

#### Evaluation of the ambulatory care rotation

Both groups of students agreed to positive statements more often than to negative ones. The ambulatory care simulation, however, had no influence on the evaluation of the ambulatory care rotation (see table 7 (Tab. 7)). The reliability of the questions asking for an evaluation of the rotation is α=0.90.

#### Assessment of performance in the ambulatory care setting

There were no significant differences between the CG and EG in the assessment of performance in the ambulatory care setting (see table 8 (Tab. 8)). The reliability of the statements on working in the ambulatory care setting is α=0.59 (pre-measure) and α=0.35 (post-measure).

## Discussion

### Evaluation of the teaching format for the ambulatory care simulation

Training with simulated patients should be based on clinically relevant, authentic and straightforward scenarios. These allow students to test and improve their skills in diagnosis, communication, documentation and case management under authentic conditions [12]. This study was not focused on separately evaluating the six different simulated scenarios. This approach appeared feasible since the four main learning objectives were present in each scenario and all scenarios shared the special features of working in ambulatory care. Accordingly, the roles for the SP, the teaching plans for the instructor and the information for the students all focused on the management of the simulated scenarios with regard to these learning objectives. The question of whether there are differences in the students’ evaluation of the individual simulated cases cannot be answered given the study’s design. Likewise, the question of whether there are differences in the investigated criteria between the participants who were assigned preparatory tasks and those assigned with follow-up tasks remains open (see figure 1 (Fig. 1)). Clarification of these issues could be a starting point for further investigation.

#### Subjective learning success

At the end of the rotation the students in both groups partially to mostly agreed with statements on subjective learning during the ambulatory care rotation. No statistically significant difference could be detected between the CG and EG. A reason for the same assessment of learning success in both groups at the end of the rotation could be the diverse and statistically elusive influences connected with the individual rotation placements. These offer countless learning opportunities that cancel out any differences making the variance of the measured variables larger and the effect smaller. In respect to the overall effect of a 12-week final year rotation, the educational effect of an intervention with four instructional units at the beginning can only be shown statistically in a much larger cohort. Another reason could be the time at which the second measurement was taken: at the end of the rotation, meaning about 10 weeks after the ambulatory care simulation. This was done on purpose in order to eliminate short-term effects of the educational intervention. These must be reproducibly measured in the subjective self-assessment following an intervention; however, they are of doubtful importance if no sustained learning effect takes hold. A further reason could lie in the intervention’s lack of effectiveness. In the evaluation of the teaching format for the ambulatory care simulation by students who participated, they rated statements about the quality of case structure, ease of comprehensibility, overall effectiveness, and sense of time pressure with a high degree of agreement. Conversely, the statement that the cases were too easy was largely rejected. However, the questionnaires were unable to uncover whether the selection of learning objectives and cases met the participants’ needs during the rotation even though the demands posed by the various rotation placements very possibly differ greatly. This would make it even more difficult to measure subjective learning progress as a mean value of the overall group.

#### Acquisition of competencies

At the beginning of the rotation the mean values for self-assessed proficiency in the selected competencies showed a wide dispersion in both groups (CG and EG) between the highest and lowest values. Particularly good self-assessment values were assigned to the competencies related to the role of physician as communicator. This reflects the efforts of recent years to anchor this role in the curriculum specifically: the students enrolled in MaReCuM pass through a longitudinal learning spiral throughout years 1-5 of study. They undergo courses on taking patient history and holding medical consultations as early as in their first year. During years 3 to 5, the students attend courses in a simulated patient program. The educational formats encompass small-group instruction and training with simulated patients. The curricular content is tested using a practical assessment (OSCE=Objective structured clinical examination). One OSCE station (of nine in total) for the introductory module on clinical examination in the third year of medical study tests communication skills/competencies only. In the fifth-year interdisciplinary OSCE, communication is a core component weighted at 25% for each of the 12 stations.

Especially poor values were assigned in the self-assessment to competencies related to quality assurance, prescribing medications, and assessing the incapacity to work, occupational disability and reduction in earning capacity. The deficits connected with prescribing medications in particular are corroborated by the literature. Simon R. J. Maxwell describes the prescription of medication as a complex task which requires the combination of diagnostic abilities, pharmacology, communication skills and a critical assessment of benefits and risks. Imparting this competency is understood to be a major teaching challenge [17]. Approximately 10% of prescriptions issued by young doctors are erroneous [18], [19]. This represents a serious risk to patient safety and needs to be improved from the perspective of quality assurance. Based on learning theory and concrete recommendations, an improvement in competency could be achieved through supervised simulated exercises in prescribing medications. This should be placed at the end of the curriculum and shortly before the experience of real professional situations [20], [21]. Members of the EG assessed their ability to correctly prescribe significantly higher than members of the CG. This can be viewed as an effect of the ambulatory care simulation. This effect was measured approximately 10 weeks later which speaks for a sustained effect. What cannot be answered by this study is if the students actually possess competency in correctly filling out narcotic prescription forms. Clarification of this issue remains the subject of future study.

Just as for writing prescriptions, the same learning theory indicates that the level of competency in identifying the incapacity to work, occupational disability and reduced earning capacity could also be improved by guided simulated exercises at the end of medical undergraduate study. In this study the participants in both groups assessed their competency in taking patient history, writing prescriptions, presenting patient case histories, determining and justifying the incapacity to work, occupational disability and permanent reduction in earning capacity, as well as documenting medical decisions to be significantly higher at the end of the rotation than in the beginning. It appears that strategies in healthcare and preventive medicine and knowledge of patient management can be sustainably taught in the context of ambulatory care. This also applies to dealing with social, financial and ethical aspects of diseases [4], [6], [7], [8], [9], [10].

#### Evaluation of the rotation in ambulatory care and working in the outpatient setting

Students agreed with statements such as “I find working in a doctor’s office fun” and “I can well imagine working in an ambulatory care setting or doctor’s office as a professional goal” both at the beginning and at the end of the rotation. The teaching format of the ambulatory care simulation had no influence on this evaluation. Students found working in the ambulatory care setting to be challenging, an evaluation also not influenced by the ambulatory care simulation. This was to be expected since the participants did not have greater learning success or skill acquisition in comparison to the CG. Two questions asked participants to assess the extent to which they were successfully able to apply the knowledge they gained from the ambulatory care simulation to their daily work. Two further questions explored whether they were more satisfied with their work as a result of applying the knowledge they gained and if they felt they performed their work better. The predominant response to these four questions was a tendency to disagree. A potential explanation for this result could be the participants’ sense of themselves as medical students who are not yet fully fledged practicing physicians.

This study is subject to several limitations. Only self-assessments from the students were gathered to investigate learning progress and acquisition of competencies. An objective measure of these parameters was not carried out. This study was not able to provide a differentiated evaluation of the individual simulated scenarios. The question to which extent the individual scenarios contributed to the overall evaluation of the ambulatory care simulation cannot be answered. The participation rate in this study is with 83% generally very high; however, the response rate to individual questionnaire items was markedly lower. This affects both the CG and EG.

## Conclusion

The ambulatory care simulation had no measurable influence on subjectively perceived learning, the evaluation of the ambulatory care rotation, or working in the outpatient setting. At the end of the rotation, the participants in both groups reported having gained better insight into providing ambulatory care. At the beginning of the rotation, the participants in both groups evaluated themselves to be equally proficient in the relevant competencies. Students found the simulated patient cases to be well structured and easy to understand. The scenarios succeeded in creating an intentional sense of time pressure, which participants in both groups felt to be a typical aspect of the medical profession.

The ability to properly fill out a narcotic form was rated significantly higher by members of the EG. Participation in the ambulatory care simulation had no effect on the other competencies investigated in this study.

The final year rotation in ambulatory care can be seen as a model for teaching complex and demanding tasks in an authentic setting. The effect of only four instructional units comprising the ambulatory care simulation was not measurable due to the current form or due to the measurement point at the end of the 12-week rotation. The ambulatory care simulation will be undergoing targeted development and will be supplemented with additional learning opportunities to ensure that the main learning objectives are taught. As a result, it may be possible to see an effect on the issues investigated in this study at the end of the rotation.

## Competing interests

The authors declare that they have no competing interests. 

## Supplementary Material

Differences between the experimental and control groups regarding the NKLM learning objectives

## Figures and Tables

**Table 1:**
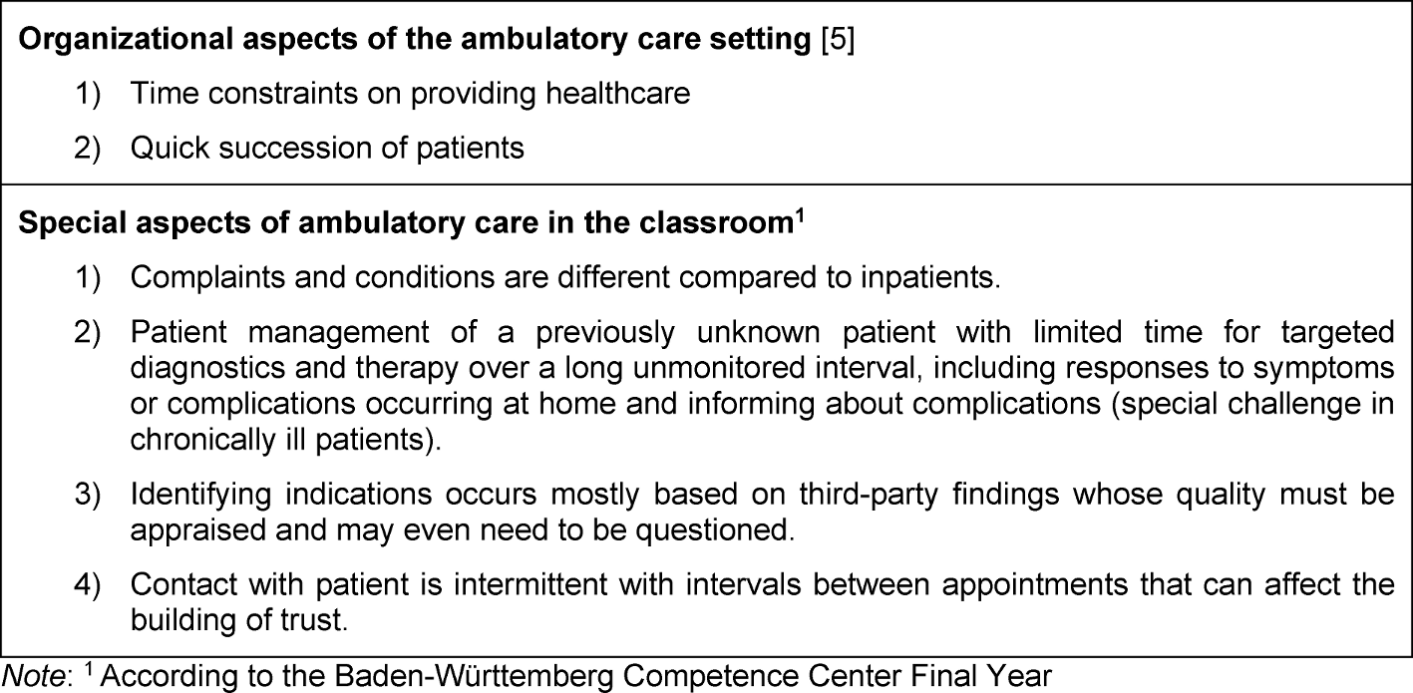
Special aspects of the ambulatory care setting

**Table 2:**
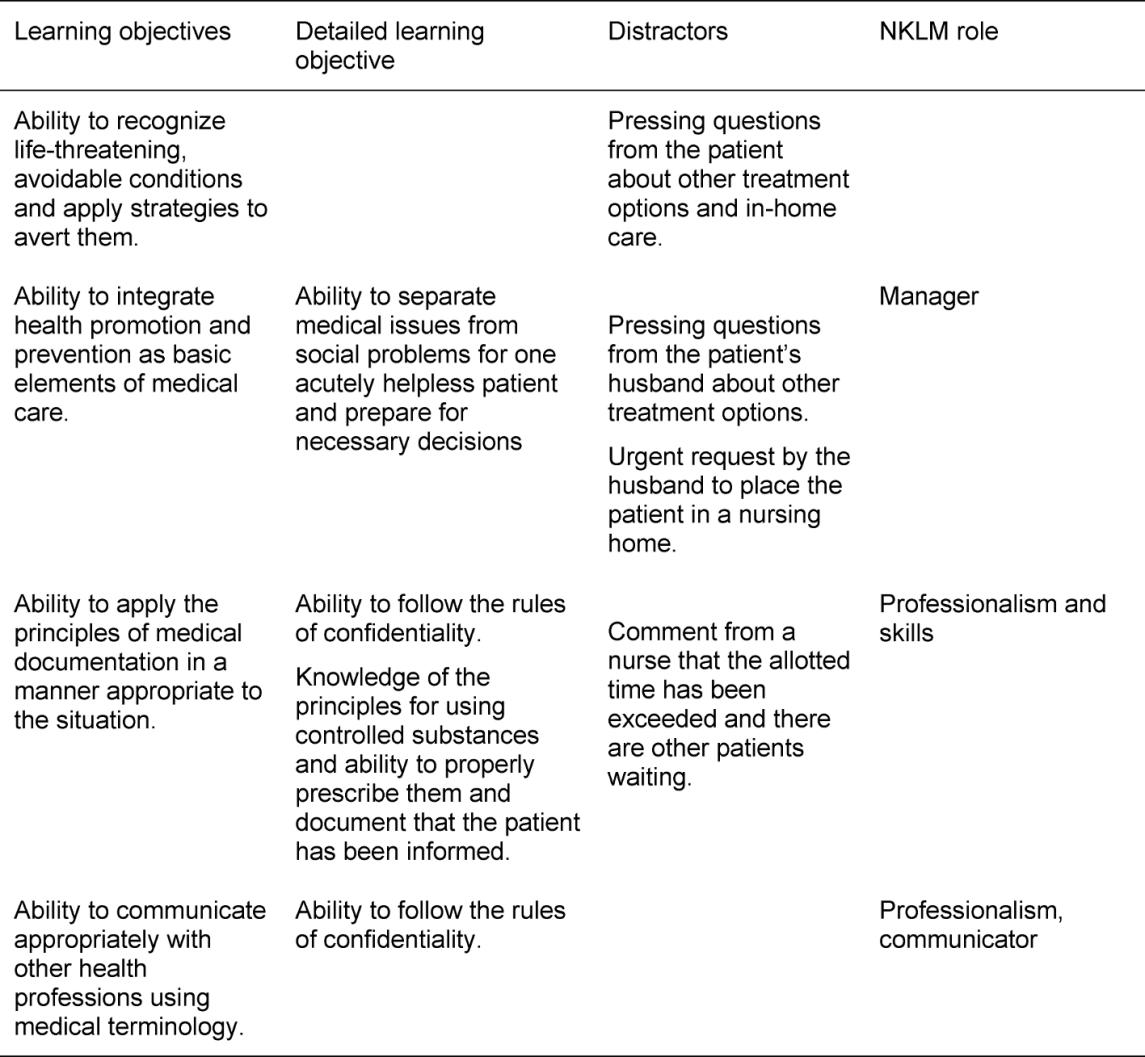
Learning objectives, distractors, roles defined by the NKLM for the case presenting myelodysplastic syndrome

**Table 3:**
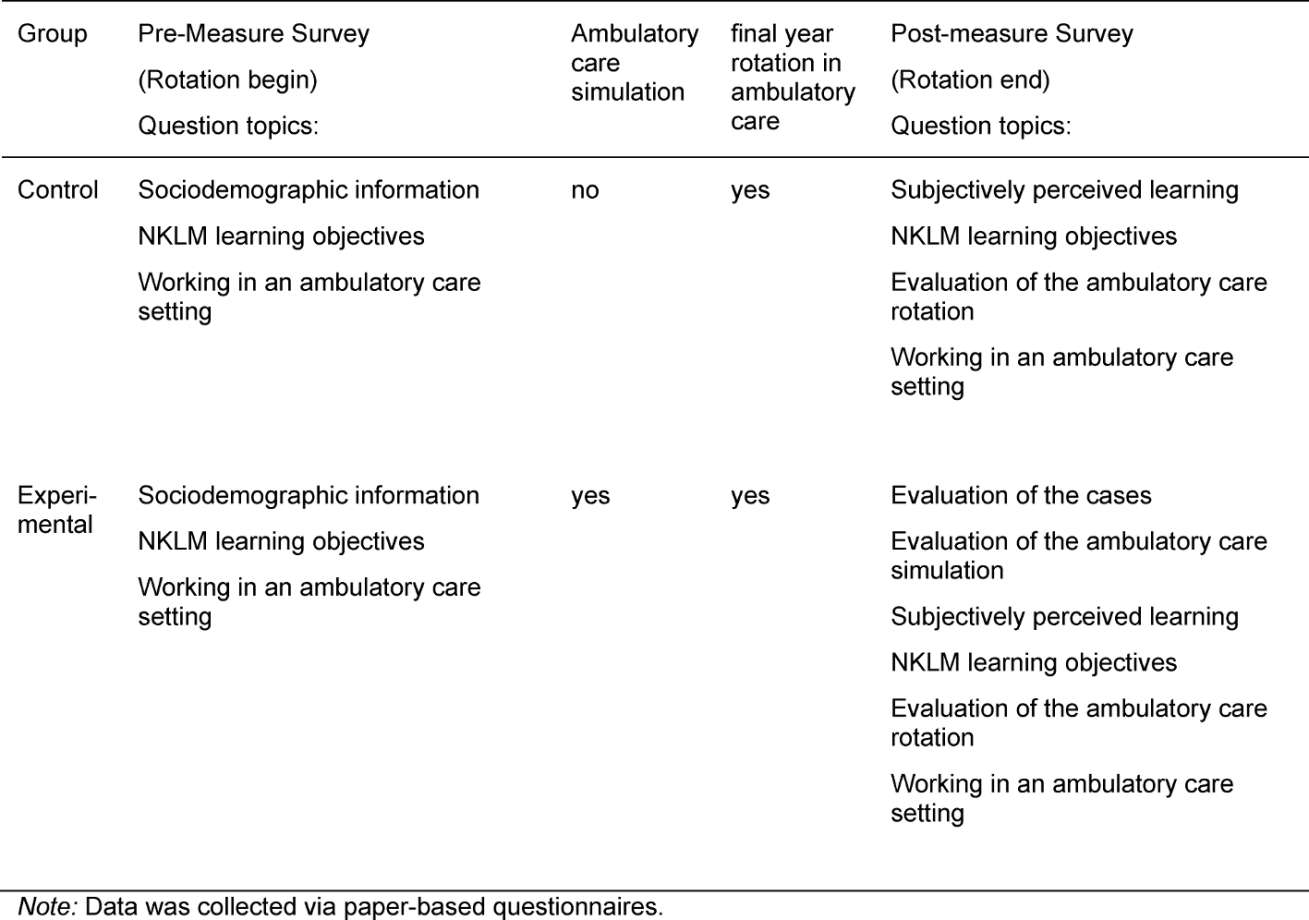
Evaluation procedure

**Table 4:**
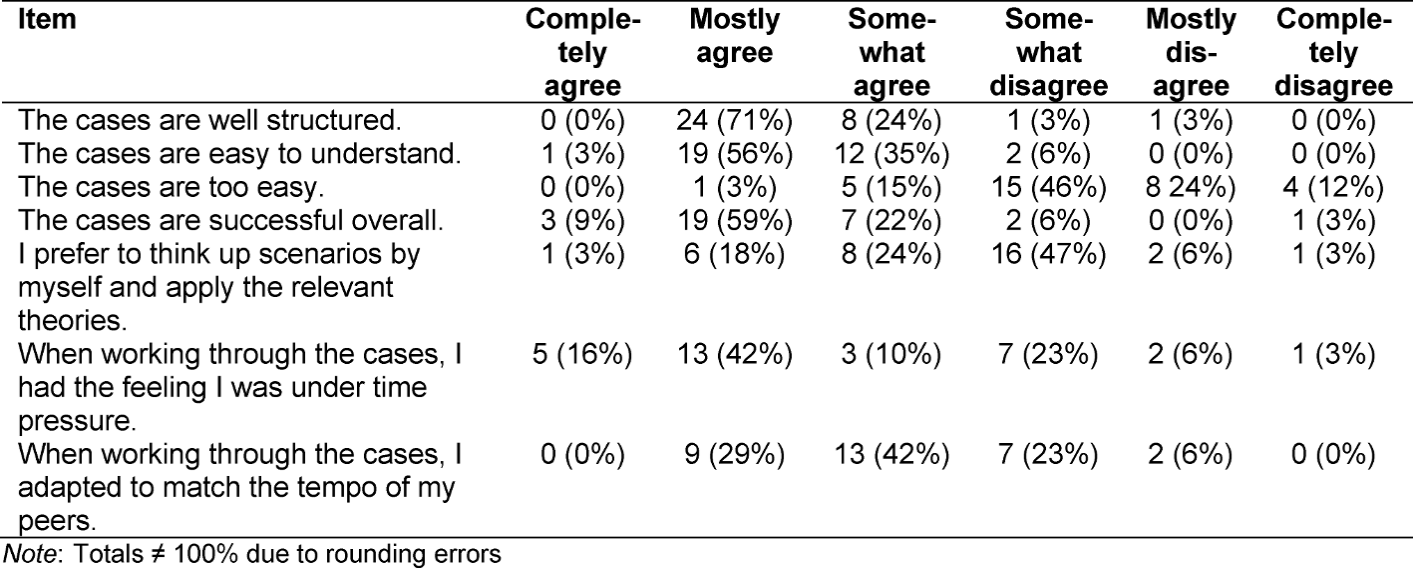
Evaluation of the simulated ambulatory care cases

**Table 5:**
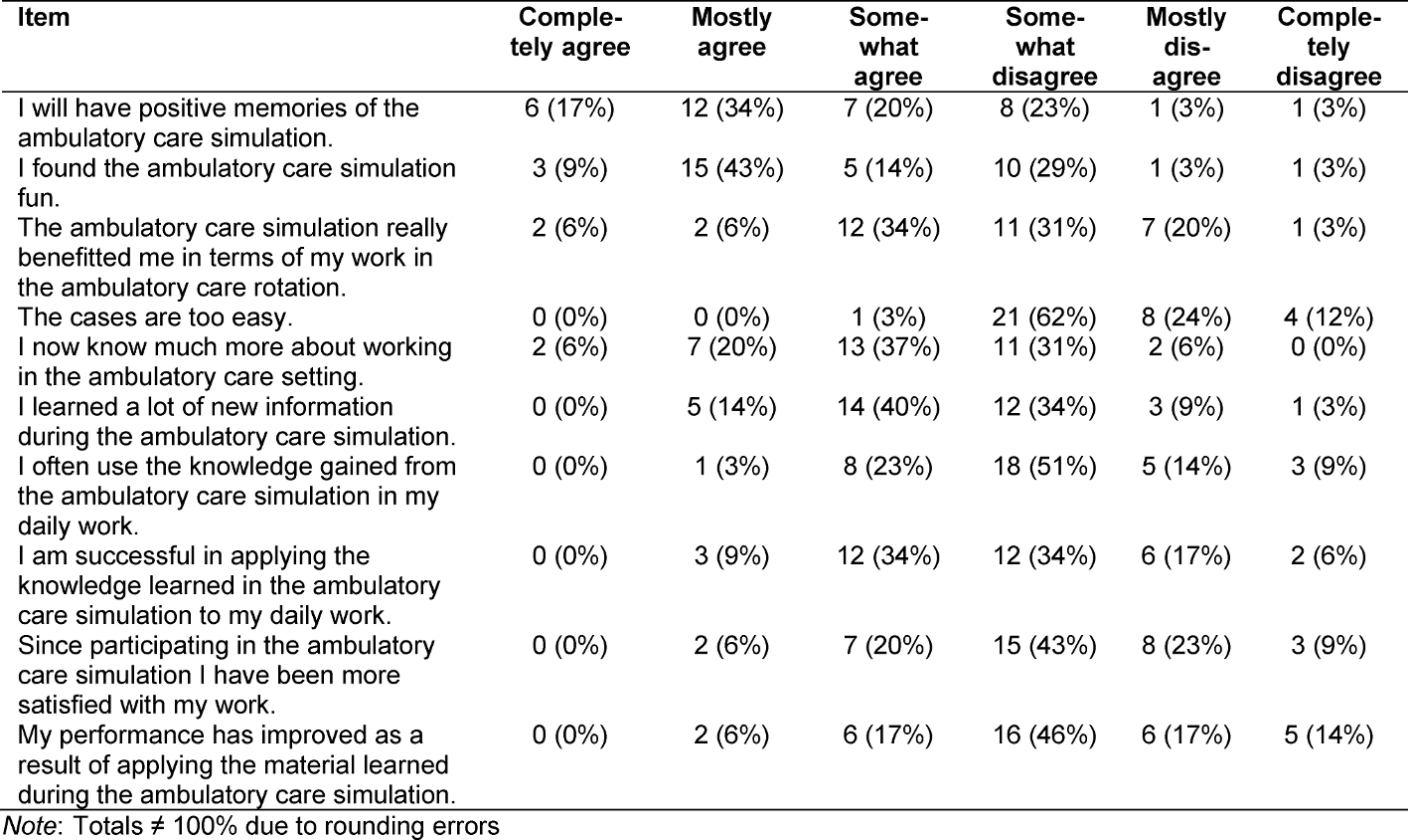
Evaluation of the ambulatory care simulation

**Table 6:**
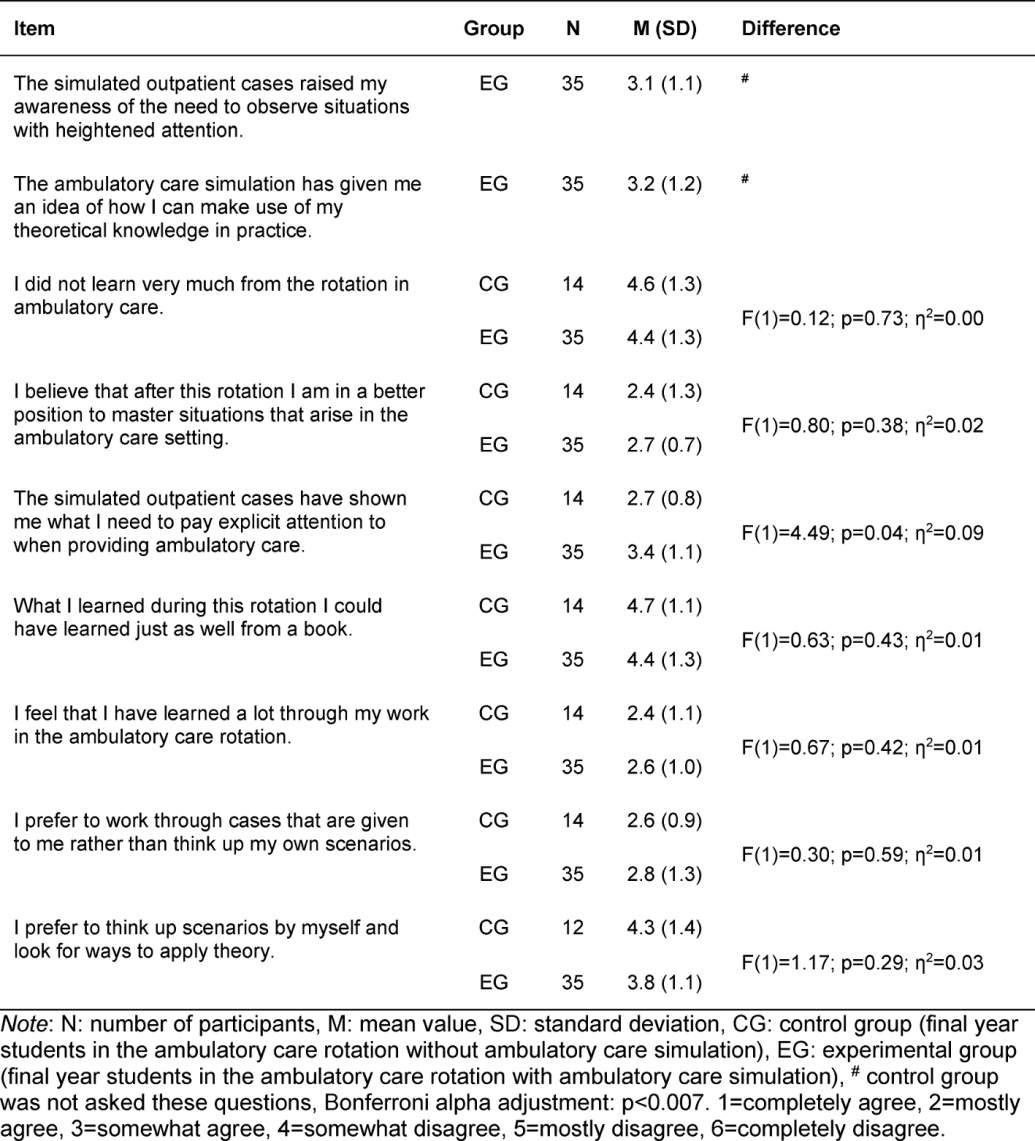
Differences between the control and experimental groups in the subjectively perceived assessment of learning at the end of the rotation in ambulatory care

**Table 7:**
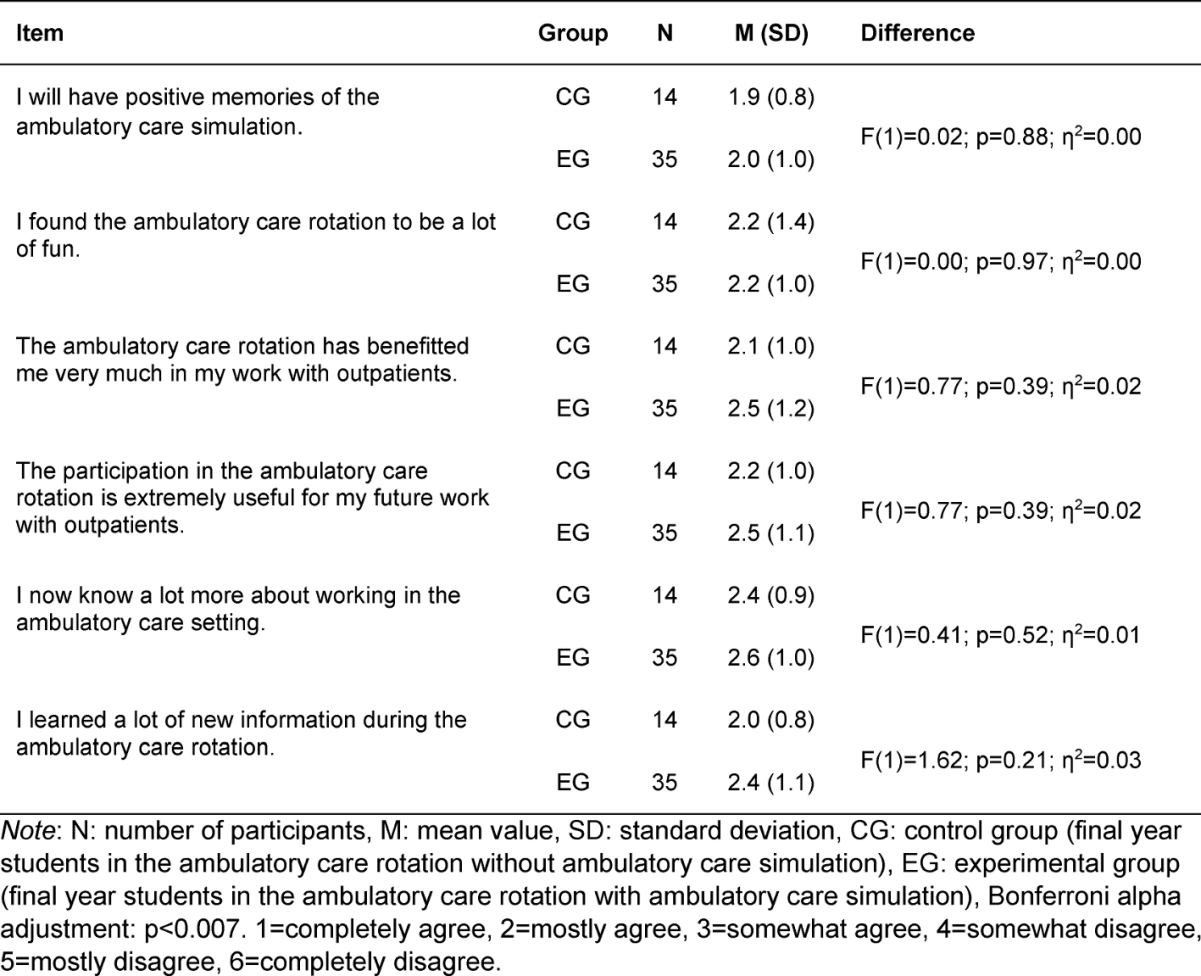
Differences between the experimental and control groups in the evaluation of the ambulatory care rotation

**Table 8:**
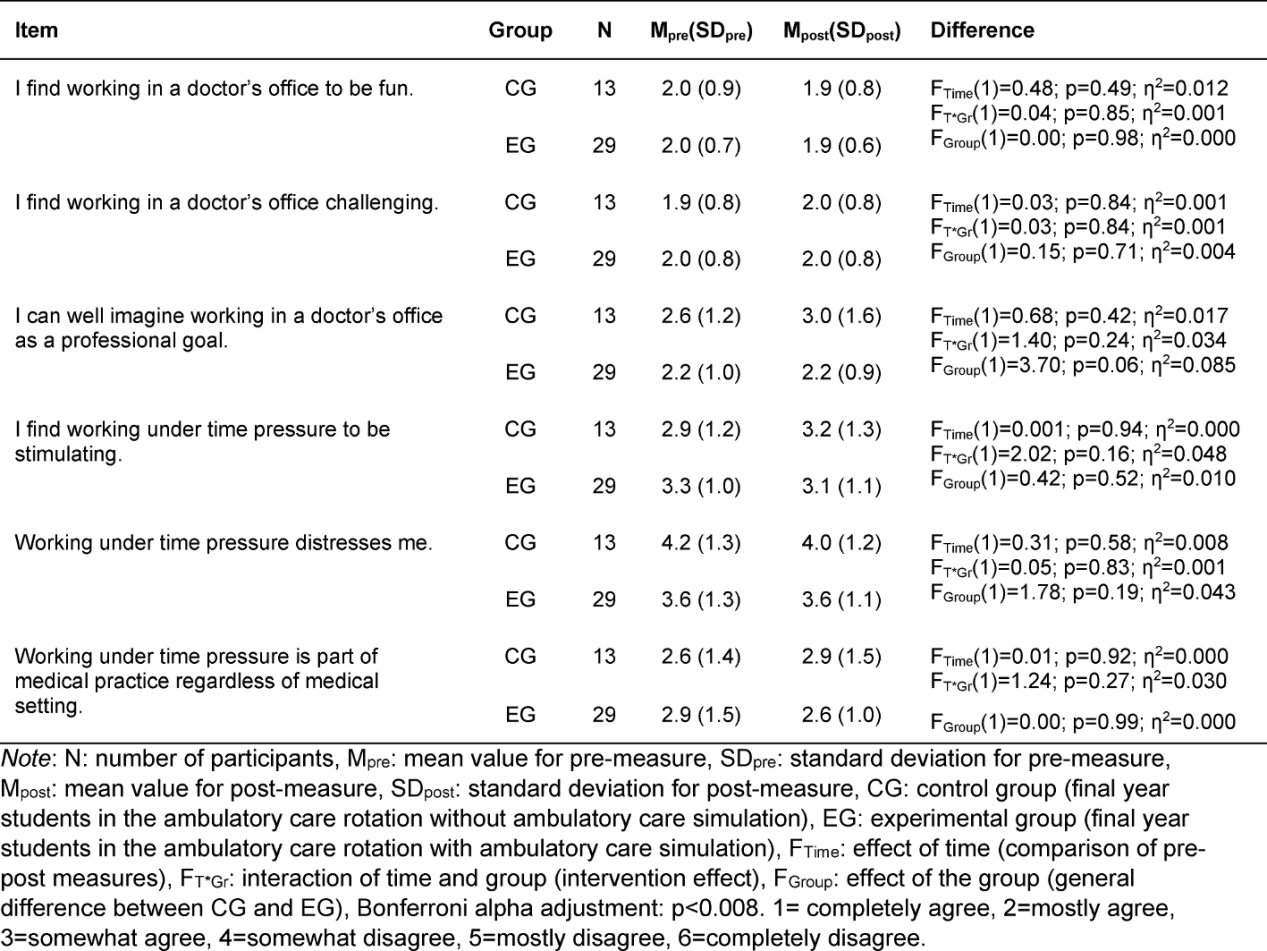
Differences between the control and experimental groups in the evaluation of working in an ambulatory care setting
