# Supplementary material for: Competency-based learning in an ambulatory care setting: Implementation of simulation training in the Ambulatory Care Rotation during the final year of the MaReCuM model curriculum
Source: GMS J Med Educ. 2018 Feb 15;35(1):Doc6. doi: 10.3205/zma001153 (PMC5827188; doi:10.3205/zma001153)
Supplement: Differences between the experimental and control groups regarding the NKLM learning objectives [file JME-35-6-s-001.pdf]

## Differences between the experimental and control groups regarding the NKLM learning objectives (Part 1)

| NKLM learning objectives | Group | N | $M_{pre}(SD_{pre})$ | $M_{post}(SD_{post})$ | Difference |
|---|---|---|---|---|---|
| Ability to hold professional consultations and apply questioning techniques in manners appropriate to a given situation. | CG | 13 | 1.9 (0.9) | 1.8 (0.6) | $F_{Time}(1)=0.48$; $p=0.49$; $\eta^2=0.01$<br>$F_{T*Gr}(1)=0.19$; $p=0.66$; $\eta^2=0.01$ |
| | EG | 29 | 2.2 (0.7) | 2.2 (0.5) | $F_{Group}(1)=4.07$; $p=0.05$; $\eta^2=0.09$ |
| Ability to give and receive feedback. | CG | 13 | 1.9 (0.7) | 1.9 (0.6) | $F_{Time}(1)=0.21$; $p=0.65$; $\eta^2=0.01$<br>$F_{T*Gr}(1)=0.21$; $p=0.65$; $\eta^2=0.01$ |
| | EG | 29 | 2.0 (0.5) | 1.9 (0.4) | $F_{Group}(1)=0.70$; $p=0.41$; $\eta^2=0.02$ |
| Ability to adequately begin interaction with the patient during consultations. | CG | 13 | 1.8 (0.6) | 1.7 (0.6) | $F_{Time}(1)=0.50$; $p=0.49$; $\eta^2=0.01$<br>$F_{T*Gr}(1)=0.01$; $p=0.92$; $\eta^2=0.00$ |
| | EG | 29 | 2.1 (0.8) | 2.0 (0.3) | $F_{Group}(1)=3.66$; $p=0.06$; $\eta^2=0.08$ |
| Ability to record a structured patient history. | CG | 13 | 2.2 (0.9) | 2.0 (0.6) | $F_{Time}(1)=14.26$; $p<0.0017$; $\eta^2=0.26$<br>$F_{T*Gr}(1)=6.25$; $p=0.02$; $\eta^2=0.14$ |
| | EG | 29 | 2.9 (0.5) | 2.1 (0.6) | $F_{Group}(1)=4.67$; $p=0.04$; $\eta^2=0.11$ |
| Ability to write adequate doctor's letters. | CG | 13 | 2.5 (1.0) | 2.4 (0.8) | $F_{Time}(1)=5.47$; $p=0.02$; $\eta^2=0.12$<br>$F_{T*Gr}(1)=3.71$; $p=0.06$; $\eta^2=0.09$ |
| | EG | 29 | 3.7 (0.1) | 2.9 (1.1) | $F_{Group}(1)=9.94$; $p=0.003$; $\eta^2=0.20$ |
| Ability to create a plan for diagnostic and therapy together with the patient. | CG | 13 | 2.9 (0.7) | 2.5 (0.8) | $F_{Time}(1)=7.11$; $p=0.01$; $\eta^2=0.15$<br>$F_{T*Gr}(1)=0.23$; $p=0.64$; $\eta^2=0.01$ |
| | EG | 29 | 3.5 (1.0) | 2.9 (0.8) | $F_{Group}(1)=6.12$; $p=0.02$; $\eta^2=0.13$ |
| Ability to properly fill out a medical prescription. | CG | 13 | 3.5 (0.9) | 2.8 (1.4) | $F_{Time}(1)=17.61$; $p<0.0017$; $\eta^2=0.31$<br>$F_{T*Gr}(1)=1.29$; $p=0.26$; $\eta^2=0.03$ |
| | EG | 29 | 4.2 (1.4) | 3.0 (1.2) | $F_{Group}(1)=2.20$; $p=0.15$; $\eta^2=0.05$ |
| Ability to involve the relevant people in the treatment and determine their position. | CG | 13 | 2.31 (1.0) | 2.3 (0.6) | $F_{Time}(1)=0.01$; $p=0.91$; $\eta^2=0.00$<br>$F_{T*Gr}(1)=0.01$; $p=0.91$; $\eta^2=0.00$ |
| | EG | 29 | 2.52 (0.9) | 2.5 (0.5) | $F_{Group}(1)=0.93$; $p=0.34$; $\eta^2=0.02$ |
| Ability to recognize mistakes and address them up in an appropriate manner. | CG | 13 | 2.31 (0.9) | 2.1 (0.8) | $F_{Time}(1)=1.76$; $p=0.19$; $\eta^2=0.04$<br>$F_{T*Gr}(1)=0.01$; $p=0.94$; $\eta^2=0.00$ |
| | EG | 29 | 2.62 (0.7) | 2.4 (0.9) | $F_{Group}(1)=2.37$; $p=0.13$; $\eta^2=0.06$ |
| Ability to collaborate professionally, respectfully and efficiently with other medical disciplines. | CG | 13 | 2.00 (0.8) | 2.0 (0.7) | $F_{Time}(1)=0.85$; $p=0.36$; $\eta^2=0.02$<br>$F_{T*Gr}(1)=0.85$; $p=0.36$; $\eta^2=0.02$ |
| | EG | 29 | 2.59 (1.0) | 2.2 (0.9) | $F_{Group}(1)=3.15$; $p=0.08$; $\eta^2=0.07$ |
| Ability to communicate with other health professionals using appropriate terminology. | CG | 13 | 2.08 (0.8) | 1.7 (0.6) | $F_{Time}(1)=5.68$; $p=0.02$; $\eta^2=0.12$<br>$F_{T*Gr}(1)=0.02$; $p=0.90$; $\eta^2=0.00$ |
| | EG | 29 | 2.31 (0.9) | 2.0 (0.4) | $F_{Group}(1)=2.30$; $p=0.14$; $\eta^2=0.05$ |
| Ability to present case histories succinctly, clearly and in problem-oriented manner. | CG | 13 | 2.46 (1.0) | 1.8 (0.6) | $F_{Time}(1)=13.17$; $p<0.0017$; $\eta^2=0.25$<br>$F_{T*Gr}(1)=1.48$; $p=0.23$; $\eta^2=0.04$ |
| | EG | 29 | 0.66 (0.8) | 2.3 (0.9) | $F_{Group}(1)=2.56$; $p=0.12$; $\eta^2=0.06$ |

*Note*: N: number of participants, $M_{pre}$: mean value for pre-measure, $SD_{pre}$: standard deviation for pre-measure, $M_{post}$: mean value for post-measure, $SD_{post}$: standard deviation for post-measure, CG: control group (final year students in the ambulatory care rotation without ambulatory care simulation), EG: experimental group (final year students in the ambulatory care rotation with ambulatory care simulation), $F_{Time}$: effect of time (comparison pre-post measures), $F_{T*Gr}$: interaction of time and group (intervention effect), $F_{Group}$: effect of group (general difference between CG and EG), Bonferroni alpha adjustment: $p<0.0017$. 1= completely agree, 2=mostly agree, 3=somewhat agree, 4=somewhat disagree, 5=mostly disagree, 6=completely disagree.

## Differences between the experimental and control groups regarding the NKLM learning objectives (Part 2)

| NKLM learning objectives | Group | N | $M_{pre}(SD_{pre})$ | $M_{post}(SD_{post})$ | Difference |
|---|---|---|---|---|---|
| Ability to integrate health promotion and prevention as basic aspects of individual care. | CG | 13 | 2.8 (0.9) | 2.2 (0.8) | $F_{Time}(1)=3.98$; $p=0.05$; $\eta^2=0.09$<br>$F_{T*Gr}(1)=6.24$; $p=0.02$; $\eta^2=0.14$ |
| | EG | 29 | 2.7 (0.9) | 2.7 (0.9) | $F_{Group}(1)=0.80$; $p=0.38$; $\eta^2=0.02$ |
| Ability to suggest suitable measures to improve the patient's overall health. | CG | 13 | 2.5 (1.0) | 2.2 (0.8) | $F_{Time}(1)=1.75$; $p=0.19$; $\eta^2=0.04$<br>$F_{T*Gr}(1)=1.22$; $p=0.28$; $\eta^2=0.03$ |
| | EG | 29 | 2.5 (0.8) | 2.5 (0.8) | $F_{Group}(1)=0.27$; $p=0.60$; $\eta^2=0.01$ |
| Ability to identify resources for and resistance to such measures. | CG | 13 | 2.6 (0.7) | 2.23 (0.8) | $F_{Time}(1)=5.44$; $p=0.03$; $\eta^2=0.12$<br>$F_{T*Gr}(1)=0.01$; $p=0.93$; $\eta^2=0.00$ |
| | EG | 29 | 2.8 (1.2) | 2.4 (0.7) | $F_{Group}(1)=0.42$; $p=0.52$; $\eta^2=0.01$ |
| Ability to name critical organizations and their services with which health promotion can be encouraged. | CG | 12 | 2.8 (1.1) | 2.2 (0.8) | $F_{Time}(1)=5.66$; $p=0.02$; $\eta^2=0.13$<br>$F_{T*Gr}(1)=2.76$; $p=0.11$; $\eta^2=0.07$ |
| | EG | 29 | 3.0 (0.9) | 2.9 (0.8) | $F_{Group}(1)=3.72$; $p=0.06$; $\eta^2=0.09$ |
| Ability to identify and coordinate all persons and healthcare structures involved in providing health care in a concrete patient case. | CG | 13 | 2.6 (0.9) | 2.6 (1.0) | $F_{Time}(1)=2.68$; $p=0.11$; $\eta^2=0.06$<br>$F_{T*Gr}(1)=2.68$; $p=0.11$; $\eta^2=0.06$ |
| | EG | 29 | 3.4 (0.) | 2.9 (0.9) | $F_{Group}(1)=4.45$; $p=0.04$; $\eta^2=0.10$ |
| Ability to point out economic problems in the context of healthcare and propose solutions. | CG | 13 | 3.4 (1.1) | 2.6 (1.0) | $F_{Time}(1)=9.23$; $p=0.004$; $\eta^2=0.19$<br>$F_{T*Gr}(1)=3.06$; $p=0.09$; $\eta^2=0.07$ |
| | EG | 29 | 3.5 (1.1) | 3.2 (1.1) | $F_{Group}(1)=1.15$; $p=0.29$; $\eta^2=0.03$ |
| Ability to make initial decisions for an acutely helpless patient while separating the medical and social problems. | CG | 13 | 2.6 (1.0) | 2.6 (1.1) | $F_{Time}(1)=1.33$; $p=0.26$; $\eta^2=0.03$<br>$F_{T*Gr}(1)=1.33$; $p=0.26$; $\eta^2=0.03$ |
| | EG | 29 | 3.0 (1.0) | 2.7 (0.8) | $F_{Group}(1)=0.75$; $p=0.39$; $\eta^2=0.02$ |
| Ability to make and justify statements concerning specific instances of incapacity to work, occupational disability and permanently reduced earning capacity. | CG | 13 | 3.9 (0.1) | 2.9 (1.0) | $F_{Time}(1)=15.31$; $p<0.0017$; $\eta^2=0.28$<br>$F_{T*Gr}(1)=1.84$; $p=0.18$; $\eta^2=0.04$ |
| | EG | 29 | 4.3 (1.0) | 3.9 (1.1) | $F_{Group}(1)=5.27$; $p=0.03$; $\eta^2=0.12$ |
| Ability to identify measures for quality assurance and act accordingly. | CG | 13 | 3.2 (1.1) | 3.2 (0.8) | $F_{Time}(1)=0.42$; $p=0.52$; $\eta^2=0.01$<br>$F_{T*Gr}(1)=0.42$; $p=0.52$; $\eta^2=0.01$ |
| | EG | 29 | 4.0 (1.1) | 3.7 (1.0) | $F_{Group}(1)=4.77$; $p=0.04$; $\eta^2=0.11$ |
| Ability to clearly and truthfully present the chances of success regarding prophylactic, diagnostic and therapeutic measures. | CG | 13 | 2.7 (1.0) | 2.9 (0.9) | $F_{Time}(1)=0.23$; $p=0.63$; $\eta^2=0.01$<br>$F_{T*Gr}(1)=2.04$; $p=0.16$; $\eta^2=0.05$ |
| | EG | 29 | 3.3 (1.0) | 3.0 (0.9) | $F_{Group}(1)=2.04$; $p=0.16$; $\eta^2=0.05$ |
| Knowledge of the duty to follow the rules of confidentiality. | CG | 13 | 1.2 (0.6) | 1.5 (0.7) | $F_{Time}(1)=0.01$; $p=0.94$; $\eta^2=0.00$<br>$F_{T*Gr}(1)=1.68$; $p=0.20$; $\eta^2=0.04$ |
| | EG | 29 | 1.6 (1.0) | 1.4 (0.5) | $F_{Group}(1)=0.85$; $p=0.36$; $\eta^2=0.02$ |
| Familiarity with legal release from the duty to remain silent. | CG | 13 | 2.3 (1.4) | 2.0 (1.2) | $F_{Time}(1)=3.67$; $p=0.06$; $\eta^2=0.08$<br>$F_{T*Gr}(1)=0.13$; $p=0.72$; $\eta^2=0.00$ |
| | EG | 29 | 2.7 (1.1) | 2.2 (1.0) | $F_{Group}(1)=0.74$; $p=0.39$; $\eta^2=0.02$ |

*Note*: N: number of participants, $M_{pre}$: mean value for pre-measure, $SD_{pre}$: standard deviation for pre-measure, $M_{post}$: mean value for post-measure, $SD_{post}$: standard deviation for post-measure, CG: control group (final year students in the ambulatory care rotation without ambulatory care simulation), EG: experimental group (final year students in the ambulatory care rotation with ambulatory care simulation), $F_{Time}$: effect of time (comparison pre-post measures), $F_{T*Gr}$: interaction of time and group (intervention effect), $F_{Group}$: effect of group (general difference between CG and EG), Bonferroni alpha adjustment: p<0.0017. 1= completely agree, 2=mostly agree, 3=somewhat agree, 4=somewhat disagree, 5=mostly disagree, 6=completely disagree.

## Differences between the experimental and control groups regarding the NKLM learning objectives (Part 3)

| NKLM learning objectives | Group | N | $M_{pre}(SD_{pre})$ | $M_{post}(SD_{post})$ | Difference |
|---|---|---|---|---|---|
| Ability to document medical activities in a comprehensible manner. | CG | 13 | 2.3 (1.0) | 1.8 (0.7) | $F_{Time}(1)=13.27$; $p<0.0017$; $\eta^2=0.25$<br>$F_{T*Gr}(1)=0.26$; $p=0.61$; $\eta^2=0.01$ |
| | EG | 28 | 2.9 (0.9) | 2.1 (0.6) | $F_{Group}(1)=5.01$; $p=0.03$; $\eta^2=0.11$ |
| Ability to fill out a narcotic prescription form in compliance with the applicable regulations. | CG | 12 | 3.8 (1.3) | 4.0 (1.5) | $F_{Time}(1)=6.47$; $p=0.02$; $\eta^2=0.14$<br>$F_{T*Gr}(1)=13.23$; $p<0.0017$; $\eta^2=0.25$ |
| | EG | 29 | 4.7 (1.2) | 3.3 (1.3) | $F_{Group}(1)=0.15$; $p=0.70$; $\eta^2=0.00$ |
| Ability to recognize life-threatening, avoidable conditions and apply strategies to avert them. | CG | 12 | 2.5 (0.8) | 2.2 (0.9) | $F_{Time}(1)=2.58$; $p=0.12$; $\eta^2=0.06$<br>$F_{T*Gr}(1)=0.14$; $p=0.71$; $\eta^2=0.00$ |
| | EG | 29 | 2.9 (0.8) | 2.7(0.7) | $F_{Group}(1)=5.46$; $p=0.03$; $\eta^2=0.12$ |
| Knowledge of the recommendations made by the Standing Committee on Vaccination (STIKO). | CG | 13 | 2.8 (1.2) | 2.8 (1.1) | $F_{Time}(1)=0.17$; $p=0.69$; $\eta^2=0.00$<br>$F_{T*Gr}(1)=0.17$; $p=0.69$; $\eta^2=0.00$ |
| | EG | 29 | 2.9 (1.1) | 2.8 (0.9) | $F_{Group}(1)=0.07$; $p=0.79$; $\eta^2=0.00$ |
| Knowledge of the indications for various emergency vehicles and ability to dispatch them appropriately given the indication. | CG | 13 | 2.4 (1.5) | 2.4 (1.5) | $F_{Time}(1)=0.14$; $p=0.71$; $\eta^2=0.00$<br>$F_{T*Gr}(1)=0.14$; $p=0.71$; $\eta^2=0.00$ |
| | EG | 29 | 2.5 (0.8) | 2.6 (1.1) | $F_{Group}(1)=0.17$; $p=0.69$; $\eta^2=0.00$ |

*Note*: N: number of participants, $M_{pre}$: mean value for pre-measure, $SD_{pre}$: standard deviation for pre-measure, $M_{post}$: mean value for post-measure, $SD_{post}$: standard deviation for post-measure, CG: control group (final year students in the ambulatory care rotation without ambulatory care simulation), EG: experimental group (final year students in the ambulatory care rotation with ambulatory care simulation), $F_{Time}$: effect of time (comparison pre-post measures), $F_{T*Gr}$: interaction of time and group (intervention effect), $F_{Group}$: effect of group (general difference between CG and EG), Bonferroni alpha adjustment: $p<0.0017$. 1= completely agree, 2=mostly agree, 3=somewhat agree, 4=somewhat disagree, 5=mostly disagree, 6=completely disagree.
